# Preserving the self with artificial intelligence using VIPCare—a virtual interaction program for dementia caregivers

**DOI:** 10.3389/fsoc.2024.1331315

**Published:** 2024-02-05

**Authors:** Linda Francis, Moojan Ghafurian

**Affiliations:** ^1^Department of Criminology and Sociology, Cleveland State University, Cleveland, OH, United States; ^2^Department of Systems Design Engineering, University of Waterloo, Waterloo, ON, Canada

**Keywords:** dementia—Alzheimer disease, affect control theory, supportive technology, caregiving, emotion, self, artificial intelligence

## Abstract

**Introduction:**

Assistive technology is increasingly used to support the physical needs of differently abled persons but has yet to make inroads on support for cognitive or psychological issues. This gap is an opportunity to address another—the lack of contribution from theoretical social science that can provide insights into problems that cannot be seen. Using Affect Control Theory (ACT), the current project seeks to close that gap with an artificially intelligent application to improve interaction and affect for people with Alzheimer’s Disease and Related Dementias (ADRD). Using sociological theory, it models interactions with persons with ADRD based on self-sentiments, rather than cognitive memory, and informs a cellphone-based assistive tool called VIPCare for supporting caregivers.

**Methods:**

Staff focus groups and interviews with family members of persons with ADRD in a long-term residential care facility collected residents’ daily needs and personal histories. Using ACT’s evaluation, potency, and activity dimensions, researchers used these data to formulate a self-sentiment profile for each resident and programmed that profile into the VIPCare application. VIPCare used that profile to simulate affectively intelligent social interactions with each unique resident that reduce deflection from established sentiments and, thus, negative emotions.

**Results:**

We report on the data collection to design the application, develop self-sentiment profiles for the resident, and generate assistive technology that applies a sociological theory of affect to real world management of interaction, emotion, and mental health.

**Discussion:**

By reducing trial and error in learning to engage people with dementia, this tool has potential to smooth interaction and improve wellbeing for a population vulnerable to distress.

## 1 Introduction

Most computer applications are developed by computer scientists, sometimes using social science methods to obtain information from potential users. Seldom, however, is social science *theory* used to formulate an application. Even in the field of assistive technology for elder care, where artificial intelligence and machine learning are at the forefront, many technological interventions are designed around practical concerns with little or no theoretical input from the social sciences. The current study aims to address this gap, putting the predictive power of a sociological theory of affect and interaction to work designing a supportive technological tool for caregivers of persons with late life dementia.

The goal of this project was the initial development of a cellphone application that will support formal caregivers in their interactions with persons who have late life dementia. People with dementia lose access to the shared definitions of the situation and rely on internal definitions of self to make sense of events (Francis et al., 2020). Treating these individuals in a way that abrogates their self-definitions (identities and self-sentiments)—for example, infantilizing or medicalizing them—can generate negative interactions and distress. The Virtual Program for Care application (VIPCare) prompts users with a probable identity in the event for the person with dementia, thereby supporting that person’s self-definitions and encouraging more constructive interaction. The artificially intelligent application also learns from reactions to tailor future responses. If used by formal caregivers who are unfamiliar with the person (and possibly visiting family members who do not understand dementia), this simple application may reduce the common trial and error approach to developing constructive interactions in such situations.

### 1.1 Preserving the self in dementia

Extensive evidence indicates that persons with Alzheimer’s disease and related dementias (ADRD) retain a psychological sense of self despite cognitive decline (Caddell and Clare, 2010, 2013). Preserving this sense may be key to quality of life (Addis and Tippett, 2004; Beard and Fox, 2008), with positive relationships with others being crucial to that preservation (Kontos et al., 2017; Tolhurst et al., 2017). From the perspective of sociology, the self is built through interaction with others over the course of a lifetime (Mead, 1934; Stryker, 1980; MacKinnon, 1994). Over time, individuals’ roles and identities coalesce into an enduring profile of self-sentiments rooted in affective or emotional meanings (MacKinnon and Heise, 2010). Self-sentiments govern emotions and behavior, and persist even without cognitive support (Rodriquez, 2011; Francis et al., 2020). Validation of self-sentiments is a crucial emotional support often missing in residential care, producing psychological distress and disruptive behavior. While experienced and familiar care providers discern a person’s general profile intuitively and interact on that basis, caregivers who are inexperienced or unfamiliar may infantilize or medicalize the person, thereby undercutting his or her sense of self (Heise, 2007; Ayalon et al., 2009; Scales et al., 2017; Francis et al., 2019).

Self-sentiment profiles are measurable and associated behavior patterns mathematically predictable using Affect Control Theory (ACT), a rigorous socio-cultural theory of affective interactions between individuals (Heise, 1979, 2007; MacKinnon, 1994). ACT has undergone more than 25 years of development in the sociological and psychological literature (MacKinnon and Robinson, 2014) and has been adapted to work as the reasoning engine for artificially intelligent (AI) agents (Ghafurian et al., 2022). Our research has developed an ACT-based instrument to capture the sentiment profile of persons with ADRD; in our initial design study, we incorporated this data into an artificially intelligent cell phone application that simulates and learns from human interaction. We codesigned the application with the help of staff from a high quality and well-rated nursing facility in the Cleveland metropolitan area. While the technical design process was reported elsewhere (Ghafurian et al., 2022), here we describe the research findings that informed that process. We report on the support garnered from staff insights and the construction of sentiment profiles for facility residents with ADRD, and show how the final design of VIPCare was grounded in theories of human interaction and affect.

### 1.2 Affect control theory and dementia

Affect control theory (ACT) and its derivatives are social psychological theories of social interaction and self (Heise, 1979, 2007; Smith-Lovin and Heise, 1988; MacKinnon, 1994, 2015; MacKinnon and Heise, 2010). Affect Control Theories (ACT) posit that humans seek and create situations that confirm or are consistent with, and avoid or suppress situations that disconfirm or are inconsistent with, their culturally shared fundamental sentiments about elements of the social world and personally held sentiments regarding the self. Fundamental sentiments regarding the social world and the self can be thought of as our “gut level” understanding or feelings about the basic elements of social interaction (e.g., identities, behaviors, settings, and emotions) and who we really are.

Fundamental sentiments, including self-sentiments, can be measured in terms of three fundamental dimensions of affective meaning: Evaluation (e.g., good or bad), Potency (e.g., powerful or weak), and Activity (e.g., active or inactive). Each concept (e.g., identity, behavior, or self) carries its own unique EPA profile and can be located within a three dimensional affective space. These three dimensions have been verified across dozens of languages and cultures (Osgood et al., 1975; Heise, 1979, 2010). Based on the three dimensions’ correspondence to a variety of other social and psychological phenomena (Fontaine et al., 2007; Scholl, 2013) ACT argues that the EPA space is a universal organizing principle of human socio-emotional life.

EPA profiles of concepts are measured using a semantic differential, on which respondents rate affective meanings of concepts on numerical scales. For example, the EPA for the identity of “nurse” is [1.7, 0.9, 0.3], meaning that nurses are seen as quite good (E), a bit powerful (P), and neither active or inactive. Bearing an EPA profile of 0.9, −0.7, −1.5, a “patient” is seen as relatively less powerful and active. Within-cultural agreement about EPA meanings of basic social concepts (e.g., patient or nurse) is typically high, and EPA ratings from as few as thirty survey participants have been shown to be extremely stable over extended periods of time (Heise, 2010). Over the course of a lifetime, individuals also develop a fundamental and enduring understanding of who they are in terms of their own relative goodness (E), potency (P), and activation (A) (MacKinnon, 2015). These fundamental self-sentiments are believed to facilitate and reflect the selection and enactment of particular social identities (MacKinnon and Heise, 2010).

Social events cause transient impressions of identities, behaviors and self that deviate from their corresponding fundamental sentiments. ACT models this formation of impressions from events with a minimalist grammar of the form actor-behavior-object. Consider, for example, a nurse (actor) who ignores (behavior) a patient (object). Observers agree, and ACT predicts, that this nurse appears less nice (E), and less potent (P), than the cultural average of a nurse. The Euclidean distance between the transient impressions and the fundamental sentiments is called deflection—a state of mind that individuals seek to resolve. ACT predicts that when someone who sees himself as good (E) and powerful (P) – e.g. a former Judge or Breadwinner—is ignored or condescended to by someone who they perceive as less powerful (e.g., a Nurse), which is often the case in residential treatment centers for persons with AD (Beard and Fox, 2008; Rodriquez, 2011; Beard, 2016), he is likely to feel anger, frustration, resentment, and eventually depression. Such disconfirming interactions may also prompt disruptive behavioral changes in order to compensate for the experience of being overlooked or devalued (Kroska and Harkness, 2008; Konig et al., 2017).

We build on previous work with BayesACT (Schröder et al., 2016), which is a probabilistic and decision-theoretic generalization of ACT, that has demonstrated how it can be leveraged to build affectively intelligent artificial agents. BayesACT expands on ACT by enabling us to generate affectively intelligent interactions with people based on their fundamental self-sentiments with an added element of uncertainty, predicting a set of potential behaviors using the affect control principle, which informing actions that are simultaneously goal-directed and affect-sensitive. Drawing on a growing body of research on dementia-friendly applications (Sposaro et al., 2010; Welsh et al., 2018), user-centered design and development methods, and using BayesACT, an updated version of the software INTERACT (Heise, 2016; Schröder et al., 2016), we are developing a simulation-based cellphone application to help providers interact with persons with ADRD in ways that work with each person’s self-sentiments (MacKinnon, 2015; Konig et al., 2017). We codesigned (Nielsen, 1993) this application with the assistance of staff in a highly rated residential nursing facility for adults with ADRD (Ghafurian et al., 2022). The goals of the study were to develop and assess the implementation of this tool. In the current paper, we report on the process of developing the application’s use, constructing the sentiment profiles, and programming these data to create the working version of the application.

## 2 Methods and data

### 2.1 Methodology

The research site was the dementia unit of a residential nursing facility we call “Twin Elms,” located in the suburbs of a large, midwestern city in the U.S. The facility is mid-sized with 106 beds total, twenty-nine devoted to the memory care wing. There are nineteen regular staff (nurses and nursing assistants) assigned entirely to the wing, plus a large number of part time “floaters” who work wherever assigned in the facility as needed to ensure the required number of staff on the floor each day. Twin Elms is one of a very few remaining family-owned (rather than corporate owned) residential facilities and is also one of the most highly rated facilities in the region. While rapid turnover and burnout is a common problem in residential care (Collier and Harrington, 2008), Twin Elms has some staff that have been there 20 years or more. The difficult conditions of the pandemic increased turnover and the low unemployment rates of the early 2020’s have made low-paid nursing assistant work less attractive, however, and even desirable facilities like Twin Elms are feeling the squeeze.

Sixteen staff members participated in the focus groups and co-design sessions, including three overnight staff and three “floaters.” All focus group participants were female, ranging in age from early twenties to late forties, three were African American and the rest white. Four were registered nurses, one was an occupational therapist, and the rest were certified nursing assistants. All staff members signed informed consent forms. The study team collaboratively designed the format of a virtual interaction application with nursing staff to simulate their interaction with residents with dementia based on that resident’s self-sentiment profile, regardless of cognitive status (Ghafurian et al., 2022). Staff feedback also shaped the content of the application and of the family interviews.

Conducting focus groups with nursing facility staff faced a number of constraints. Many nursing assistants depended on public transportation and could not attend outside of their normal shift hours. Running groups during work hours limited the length of each group. Safety rules requiring a minimum number of staff on the floor at all times meant that groups could not meet the normal standard number of 6–10 for a typical focus group. To accommodate these restrictions, we developed a model that we called *in vivo* focus groups: groups were each only 25–45 min long but ran sequentially over each afternoon. Beginning late afternoon when residents were least active, and running through shift change (6,00 pm), two members of the research team ran a series of eight groups over three days in the staff lounge. This timing enabled us to include both day and evening staff (a ninth small group was run at midnight one night to include third shift staff). Because of the easy accessibility to the Common Room for the residents, staff easily were able to drop in and or leave as they were available or needed elsewhere. There is, therefore, no concrete number for how many people participated in each group. At different points, groups could be as small as two or as large as five. While such a model lacks reliability (no two groups were exactly the same), it achieves the validity of reaching the people least often consulted in dementia care: the front-line workers who work directly with the residents on a daily basis.

In this model, only the first two of the nine groups were fully focus groups responding only to interview questions. The focus of the remaining seven groups was shifted gradually from answering questions to increasingly participating in the paper prototyping (i.e., creating the layout of the application using paper, pen, stickers, etc.) as part of the co-designing the format of the application The codesign process was reported elsewhere (Ghafurian et al., 2022), while the insights from the focus group questions and the process for creation of sentiment profiles are analyzed here.

In the second phase of the study, researchers conducted semi-structured interviews with close family members to obtain the resident sentiment profile information. Residents of the Twin Elms dementia wing were in their late 80s or older, many were quite frail or ill, and most had at least moderately advanced dementia. At the time of data collection, one resident was Puerto Rican, and the rest were white. Characteristic of the history of the Midwestern U.S. city where Twin Elms was located, most of the residents were ethnically Eastern European, usually second generation (their parents were immigrants). Most had followed very traditional gender role divisions: all had been married, men had been breadwinners and women homemakers. Most family members interviewed were spouses or adult children, though one was a sister-in-law and another a sister of residents with no children.

There were 26 residents on the wing at the time, but five had ADRD that was too advanced for any interaction at all, and one was very ill and receiving hospice care, and their families did not participate. The nursing facility sent invitation letters to families of the 20 eligible residents and family members who responded were scheduled for interviews; a total of 13 families participated in interviews. Interviews were initially in person but were transferred to phone or videoconference during the COVID-19 pandemic. Whether in person or remote, all family members signed informed consent forms permitting use of their de-identified data for research purposes. Interviews with family caregivers lasted 40–90 min and were recorded and transcribed; details about residents (e.g., personal and place names or identifiable events) were deleted or modified to protect confidentiality.

Note that obtaining sentiment information from family members is not the usual approach used by ACT researchers and is not ideal. A better option would have been able to ask the residents of Twin Elms directly about their self-sentiments, measuring them on a semantic differential scale typical of ACT. Unfortunately, most residents at Twin Oaks were not able to complete such scales. In addition, the continuing COVID-19 epidemic created ethical concerns about having outsiders in close proximity to vulnerable individuals for the amount of time needed. However, using caregiver estimates of resident sentiments was also problematic. A prior study (Francis et al., 2020) collected semantic differentials on the self-sentiments of the person with dementia and did so from both the persons themselves and their family caregivers. We found only moderate agreement between the person’s own ratings and the ratings estimated by the caregiver, rendering largely unusable data. Caregiver bias tended to reflect the family role of the person with dementia (e.g., mother, husband). Due to this limitation, we chose not to collect actual resident self-sentiments from family caregivers directly via semantic differentials. Instead, we collected reports of identities, traits, and adjectives from family members’ interview descriptions. EPA ratings for these descriptors were then obtained from the 2015 U.S. dictionary (Smith-Lovin et al., 2016) to estimate self-sentiments. While this does not perfectly capture the global self-sentiments of the individual (MacKinnon, 2015), it creates a proxy of the situational self-sentiments for the identity being enacted. This approach is less sensitive than the use of semantic differentials but it minimized the risk of caregiver bias for the purposes of this initial study. The resulting information was programmed into VIPCare for each resident to produce a probability distribution of identities (Schröder et al., 2016) from which the application could select.

### 2.2 Sentiment profile coding procedure

The virtual interaction tool employs a probabilistic model of interactions involving multiple social identities and attributes simultaneously (Schröder et al., 2016). This model of affectively intelligent social interactions informs an application to model for residential care providers the most effective interaction strategies for the needs of each resident. For the application to simulate such interactions, the application needs to have the identities and behaviors of the actor (the caregiver) and the object (the resident) to create an actor-behavior-object (ABO) event. Resident identity EPA ratings were programmed into VIPCare as probabilistic sentiment profiles for each resident. Behaviors were identified through a menu of the key activities staff engage in with residents (see Ghafurian et al., 2022). These included behaviors such as eating, getting dressed, taking medication, etc.

A remaining challenge was populating the identity of the object (caregiver) identity. Just as persons with dementia lose access to shared definitions of the situation, it also becomes more difficult for others to take the role of persons with dementia and see ourselves through their eyes (Cooley, 1902). We initially considered fixing the object for all interactions in the role of “nurse,” as most staff at Twin Elms could fit some variation of this identity. However, while some resident identities might make situational sense interacting with a nurse (e.g., mother and nurse, or teacher and nurse), others seemed more likely to spark deflection (e.g., factory supervisor and nurse). For this early version of the application, therefore, object identities for the caregiver/user are chosen as complementing the resident identity. For example, if the resident identity is “teacher,” the object identity is “student.” These identities are programmed into VIPCare based on those most definitional to a role. By definition, “mother” requires a “child” and “employer” requires an “employee.” This creates challenges for identities that are not based in institutional roles, however; examples described in the results include “homebody” and “immigrant,” which are not so easily matched. Currently, the strategy is to duplicate the identity for the actor (caregiver), such that an immigrant will interact with another immigrant. Future plans to expand object identities are set forth in the discussion.

We used the ACT dimensions of evaluation, potency, and activity to encode sentiment profile scores. To do this, researchers compiled from interviews the documented identities and attributes of residents, gathered their corresponding EPA profiles from existing sentiment dictionaries, and created an overarching self-sentiment profile. Specifically, we used the extensive ACT dictionaries of cultural sentiment ratings (Heise, 2010) that contain thousands of identities, traits, and moods, each one rated on the dimensions of evaluation (E), potency (P), and activity (A). These dictionaries have been collected over the course of more than 35 years, in seven countries, from thousands of respondents. They have also been validated using an array of qualitative and quantitative methods (MacKinnon and Robinson, 2014).

The sentiment profile interviews with the family caregiver were coded for the core building blocks of the resident’s sense of self, − that is, the identities (e.g., mother, veteran), traits (e.g., generous, stubborn), and prevailing moods (e.g., cheerful, depressed). These were recorded and weighted by the significance attached to them by the respondent. Salient identities, characteristics, and moods were considered most reflective of the resident’s core sense of self. Using the ACT dictionary of cultural sentiments, we assigned each resident’s most heavily weighted identities, traits, and moods a three-number EPA sentiment rating. Since residents will each have more than one core identity, trait and mood, each person will have multiple ratings. We compiled these ratings as weighted distributions of sentiments for core identities, traits, and moods for each resident. A profile “score” therefore, is effectively a three-cell table, each cell containing a set of sentiments and weights corresponding to the resident’s reported sense of self. The computer science researchers then programmed the BayesACT-based simulation tool to be tailored to each resident on the unit. The identities cell currently serves as the main driver of the simulation program; as the application develops we will also program into the application resident traits and moods as key adjectives used to modify those identity sentiment ratings.

## 3 Results

### 3.1 Results: focus groups

The goals of the focus group interview questions were (1) to elicit the information that staff saw as necessary for people to have in order to be able to interact effectively with residents, and (2) to identify factors that produce negative interactions with residents (e.g., make them agitated, upset, confused, etc.). For the first of these goals, staff consistently said that the most essential tool was knowing the person well enough to predict their reactions. When asked how staff learn to work with residents (e.g., new staff members learn to work with residents or current staff learn to work with new residents), the first requirement was to learn about the resident.

[Y]ou have to observe. You cannot treat every resident the same, you have to learn them and that can help you determine how to be with that resident.

You have to KNOW who you are working with before you can even do the task, because if you do not know how to approach that person you can run into a problem right then and there. And if you agitate them as soon as you interact, then you can probably chonk it. [laughter]. You can chonk it, and you are going to have to wait some time and see if they calm down. And they may even not want to deal with you for the rest of the day. So yeah, you have to know the person first before anything.

Another nurse describes how this need to know the resident is built into staff training.

One of the things, in the classes I’ve taken, some of the questions were: what do they like to eat? What’s their favorite smell? Like if they love the smell of lilacs. You really want to know what makes them angry or sad or something because you want to avoid that. Stuff like, “oh, tell me about your children,” but [not] if that’s something that’s not a good memory. Or if they were really into cars….

Families were also a good source of information about residents, but one staff member pointed out that even they sometimes left out information that staff would like to know.

[S]ometimes the family will give us some information. But a lot of times you are begging for the information [laughter] and you just got to find out on your own. Like eventually you find out where they used to work…but sometimes it’s like in their obituary you find out a lot of things that you had no idea and you have been taking care of this person all this time. And they had a fascinating life you did not know about. So that’s sad.

The above quote also hints at one of the key kinds of information staff wanted to have: they wanted to know the residents’ past identities. For example, when discussing what kind of information they give new staff, one nursing assistant said,

Something like what was on the example, where it notifies you that the resident was an army officer. So like you would kind of know their background, and that would give you a little bit of a heads up about how to approach them, because if he’s an army officer he’s used to being in control, he’s not going to take too well to somebody ordering him around.

And doctors too [laughter].

Or if they were priests or anything like that, yeah.

You have to kind of know their background to know the approach.

The staff returned to this point a few minutes later, reiterating the importance of identities, especially work identities for people with dementia.

Prime example: Bonnie. She was a teacher, so she is very firm. She is the boss. I mean she IS the boss. When she gets in her moods you’ll hear her ordering the other residents around, ordering me around, it’s so funny, little things…. So like she’ll hear a bathroom call going off and she one time thought that it was somebody disrupting class, playing with the bell. And she was like “all right, whoever’s got that bell, you stand up right now!” But definitely, if you know that someone’s a teacher, like she said, back in the day, most teachers were pretty firm.

This idea emerged in other groups as well. For example, one nurse joked about buying Bonnie some homework sheets and have her grade them. Another said,

But we have had people like the boss at the Ford plant, and Nina worked at the restaurant, and she’d always talk about Bolognese sauce, so yeah, they remember that stuff forever if it’s something they did for a long time.

This is particularly interesting because the need to know the residents’ past identities confirms the premises of this study. The description of Bonnie responding as though she is still in the classroom corresponds perfectly with the findings of Francis et al. (2020). That study illustrated how people with dementia may lose their ability to access a shared definition of the situation (Mead, 1934), because that requires access to cognitive cues. However, even in cognitive decline, people are able to access their internal self-sentiments as affective cues and can use those to define the situation and shape their behavior. Relying on internal affective cues, however, can often lead to the person with dementia acting in ways that are out of sync with the situation others perceive. As these nursing staff have informally learned, knowing who a person was (their identities) is an important window into understanding those sentiments and the affective cues that are driving the resident’s behavior.

Interestingly, staff pointed out that knowing a person well in the past does not necessarily translate into being able to interact with them well now that the person has dementia. When asked who could use an application like VIPCare to help them interact appropriately with residents, multiple groups mentioned the family members.

Because a lot of family members, because they do not understand the disease process, they can be standoffish, or get frustrated themselves because they are expecting their loved one to still be who they were before the dementia. So if you set it up that way [set up the app with identities] then they’ll know “okay, this is where her mind is today.”

In another group, staff pointed out that visitors (usually family) can often upset the resident with their outdated expectations. Some will continue to try to correct the resident and get them to try to interact based on a shared reality rather than the resident’s internal cues. Others will say things that show their lack of understanding of dementia and how it affects memory.

Especially when they are new, they do not know how to talk to the people. It’s like they come in after not seeing this…person for a year and it’s like “Oh, hi! Do you remember me?” And then [the residents] feel stupid because they cannot remember who this person is.

From the perspective of staff, then, knowing both the current status of the person *and* their history and biography were essential to provide the basis for effective interaction with residents.

### 3.2 Results: family caregiver interviews

To fill in residents’ histories and biographies, the research team conducted semi-structured interviews with family caregivers of participating residents. These interviews targeted two content areas: the resident’s identities and attributes (e.g., personality traits and dominant moods), and the interactional issues with staff that resulted from miscommunication or lack of familiarity with the resident at the facility.

All caregivers were able to describe their family member’s background in great detail, providing historical windows into the kind of person the resident with ADRD felt themselves to be.

I think if anybody were to ask [what kind of person he was]: kind, big heart, gentle gentleman. He was, I was divorced when my kids were three and nine…but my father was like a dad to these boys, and they grew up emulating their grandfather. So, hard worker, worked a lot of overtime. A pleaser or a people pleaser you know, beautiful voice, civically involved…in the community, Knights of Columbus and anything that was related to community. He was active at …their home parish…. He was involved in the neighborhood helping out not so much in any organized groups. He was a blood donor. He gave all the time. They were involved at [my sister’s school]…for the handicapped and it’s a school that’s modeled all across the country. It’s amazing and it’s worked for my sister. They were really involved in often levies you know for passage of bond issues. [I]f my mother had one gripe it was “Jack, when are you ever home?

I: If you could choose the top one role that had the most impact on his life, what do you think it would be?

R: Well, I know when you talk about family he will remember us, but [the auto plant], he’s at work back there. He got so much satisfaction out of that, and then I would say making jewelry. Those are all areas that I hear him talk about….

As described by his daughter, Jack in the quote above was a dedicated family man who worked hard and helped his wife raise their two daughters, one with Down Syndrome. When he was not working at the auto plant (where he rose to be supervisor) or volunteering, he made jewelry in his basement workshop. From his daughter’s description, we inferred Jack’s overall sentiments about himself as being a very good, very strong, and active person, who often engaged in strong, good, active behaviors and actions towards others.

Drawing on the Affect Control Theory dictionary,^1^ five of Jack’s key identities are shown with their EPA ratings as examples in Table 1. The application will also be set up to permit modifiers for these identities, also drawn from the caregiver interviews.

**Table 1:** Identities with EPA ratings and interpretations for resident “Jack”.

Identity	Evaluation	Potency	Activity	Interpretation
Factory supervisor	1.04	1.99	1.38	Slightly good, rather powerful, slightly active
Father	2.17	2.14	0.92	Quite good & powerful, very slightly active
Grandfather	2.38	1.22	−0.91	Quite good, slightly powerful, very slightly inactive
Jeweler	1.25	0.38	−0.28	Somewhat good, neutral power, neutrally inactive
Catholic	0.59	0.40	0.05	Neutral on all three dimensions

For Jack, such modifiers would be “hardworking supervisor” (1.78 2.00 1.87), “gentle father” (2.78 0.72–0.48), and “creative jeweler” (2.07 0.93 0.40), and “generous Catholic (1.52 0.95 0.15), each shown with how the adjectives modify the EPA ratings of the original identities. Note that only Jack’s work identity is an active identity, the rest are somewhat powerful and very good, but neutrally active leaning to inactive, though less so than the unmodified identities. So his reactions to interactions would likely change substantially depending on the identity he is enacting.

While this profile is built from one person’s perspective and a daughter certainly has a different view than, for example, the people he supervised at work, her description of his preferred activities provides a useful indication of his self-sentiments. Chances are, when Jack thinks of himself, his sentiments are more likely to agree with his daughter’s views than with those of his work subordinates.

A very different person was Annie, a widow with no children and no living siblings; her sister-in-law has charge of her care and described her for the interview:

[My sister-in-law] was, a loving person, very caring, and um thoughtful. You know like I said, she was always making sure everybody was taken care of.

I: Okay. Including just you and your family and the boys or her siblings too?

R: Well, her siblings too. She helped her sister a lot. You know the one that just died. She did a lot for her… the one sister had a lot of, she had health issues and money issues and um she helped her financially a lot. Because [her sister] was struggling a lot.

[But], I would say she was more passive than active. She…would go to the store. She went to the beauty shop once a week, get her hair done. She would go to [get groceries]. Um she did not do a lot of driving. She did not like to drive. My brother drove everywhere. Um I do not know; I’d say she’s probably middle of the road. You know, I mean if something had to be done, she was right there to help. And you know as far as her home and what she had, you know, she took charge of that. She did fine.

Here we see a woman who seems to view herself as a good person, one who wants everyone to be taken care of. But she only shows any power and activity when taking care of others or her home. Outside of home and family, she appears to be someone who views herself as weak and inactive; having few interests outside of the home and not wanting to take charge of driving herself. This quote is supported by other comments about Anne doing everything with her husband, not going out much except by themselves to their rural cabin, and her devotion to her home, cooking, and garden. Again, this is based on the report of a single person, but the activities described are illustrative. Five key identities derived from this interview are shown in Table 2.

**Table 2:** Identities with EPA ratings and interpretations for resident “Annie”.

Identity	Evaluation	Potency	Activity	Interpretation
Wife	2.92	1.23	1.03	Very good, slightly powerful, and slightly active
Sister	1.81	0.66	0.67	Somewhat good, very slightly powerful and active
Daughter	2.33	0.29	0.99	Quite good, neutrally powerful and slightly active
Homemaker	1.98	0.25	0.89	Somewhat good, neutrally powerful and slightly active
Cook	1.73	1.02	1.82	Somewhat good, slightly powerful, and somewhat active

For Annie, modifiers show her as “affectionate wife” (3.00 0.99 0.32), a “caring daughter,” (3.21 0.96 0.43) a “helpful sister,” (2.62 1.08 1.18) an “organized cook,” (2.27 1.16 1.09) and a “passive homemaker” (0.62–0.99 −0.92). Note that with the exception of adding “passive,” these modifiers tend to make her more good, more powerful, and more active. That means that if these are who she feels she is, then confirming these sentiments would make her better than just confirming the identity without the modifier, thereby making the application more accurate.

Interviews also highlighted problems with interaction at the nursing facility, especially when the resident had just moved there. In the quote below, Henry’s wife describes how he did not take on the identity of “patient” easily when he first moved to the facility:

I do not know how much you have done with Dementia but they are very anxious you know…. He almost passed out with a shower once because you know depending on who’s taking care of you some people are able to jolly you into it and say well it’s time for a shower and grab you but there is one or two that get impatient with you because there are six other people there to do….

I: So, he does not like people taking care of him…?

R: …He likes to do his own thing. He likes to be taken care of, you know, he’s pretty bossy as he is a little self-centered but no, he does not like to be pushed, prodded, or forced to do so many things. So, when I go to feed I moved his food around and these other times when he’s not as alert then he’ll let me feed him but yeah,… I know the nurses said I think he’s used to it now, but when he first got there he had to be pushed to have this clothes and his diaper put on and all that I think he was not a good patient. “It’s not that” or “leave me alone” or whatever.

From Karla’s description of her husband Henry, we derived five key identities as examples in Table 3.

**Table 3:** Identities with EPA ratings and interpretations for resident “Henry”.

Identity	Evaluation	Potency	Activity	Interpretation
Husband	1.77	1.16	0.68	Somewhat good, slightly powerful, very slightly active
Truck driver	0.99	0.85	0.81	Slightly good, slightly powerful, slightly active
Marine	1.84	0.90	1.81	Somewhat good, slightly powerful, somewhat active
Homebody or hermit	−0.08	−1.50	−2.48	Neutrally good, somewhat powerless, very inactive
Garage saler (shopper)	0.88	0.54	1.31	Slightly good, neutrally powerful, slightly active.

From the ratings of Henry’s identities, we can construe some additional detail about his reactions to staff. Most of his identities are neither particularly powerful nor active, other than his Marine role and that was far in the past. Given the homebody identity, it is possible that his annoyance in the quote stemmed from a desire to be left alone. This makes him stand out from Jack, who often enacted his powerful, active work identity and reported had difficulty adjusting to the weak and inactive identity of patient/nursing home resident. One would think it would be easier for women of that generation, who were often homemakers, but a quote from the resident Daphni’s daughter shows that such is not the case:

I: So sounds like she stayed very busy after she retired?

R: Oh yes, she wasn’t the type that would just sit around and watch tv, and I feel like that’s why she is having a bit of trouble. Sometimes I feel like she is a little too active for [the nursing facility]. Because for a while there she was just in a wheelchair and then when they moved her from rehab, she was in the wheelchair for a while, then physical therapy ended and they said to me. She’s walking, you know and I said I do not want her just sitting in the wheel chair, I said please make sure she is using the walker and once they did there was no stopping her. They were probably hoping/wishing she was back in the wheelchair; we have had some issues….[She’s] very self-sufficient…

I: Do you think that gets in her way at [the facility]?

R: I think a little bit when they are trying. And I told them she’s kind of head strong. Before they had all these alarms on her and whatever, but that would just aggravate her. So now they do not do that anymore, and they aren’t forcing her to use the walker anymore and I think she’s a little happier because of that. I mean once in a while she gets a little upset with them, but she’s doing better. And they have kind of found a medication that helps her as well with her behavior and her sleep, because she wasn’t sleeping either.

Daphni, a Greek immigrant, was very used to managing things across her life and when she had to move to the nursing facility, she did not adjust well to being managed herself. From Daphni’s daughter’s description of her in the interview, we have derived the five identities shown as examples in Table 4. Note that most of these identities are quite good, somewhat powerful, and all are more than neutrally active. These cohere well with her daughter’s description of Daphni’s behavior at Twin Elms.

**Table 4:** Identities with EPA ratings and interpretations for resident “Daphni”.

Identity	Evaluation	Potency	Activity	Interpretation
Wife	2.92	1.23	1.03	Very good, slightly powerful, slightly active
Mother	2.90	2.43	1.35	Very good, very powerful, slightly to somewhat active
Baker	2.05	0.58	1.28	Quite good, neutrally powerful, slightly active
Immigrant	0.67	−1.00	0.23	Neutrally good, slightly powerless, neutrally active
Friend	3.17	1.68	1.44	Very good, somewhat powerful, and slightly to somewhat active

In the cases of both Daphni and Henry, we see that even with cognitive decline, and even in a highly rated facility like the one in this study, residents struggle to adapt to their new situation. They no longer get to enact the roles of the identities they have held across their lives and are faced with taking on new and less desirable identities. Their experiences (and their emotional reactions) provide strong evidence of deflection caused by interaction with staff—deflection in a negative direction. The facility emphasis on getting things done (e.g., there are six other people that they need to get showered that morning) means that residents have little real control over their lives and activities. This renders them disempowered and inactive. Such disconfirmation of the self-sentiments most harbor produces deflection, and without resolution, would likely produce depression and apathy.

Ironically, in a dementia unit, the residents’ greatest protection against such an outcome is their memory loss. As long as situations like this do not become the only kind of interaction residents have, positive experiences can obscure the negative, and residents can maintain their sense of self. Returning to the focus groups, some staff gave indications of how they try to give residents control over things they can, as well as keep them active and engaged.

[W]e try to wait until they start waking up first, and then we go in there, try to encourage them to get up. Because no one wants to get up [laughter], everyone wants to stay in bed! Just encourage them to get up and go from there… you just encourage them, give them more choices, options. Like with their clothing, or what they want for breakfast; you want this or you want that. Instead of like, telling them what to do, give them more options.

Because you go trying to get them up before they are ready, that’s when they are going to become combative. Which means it sets the tone for the whole day. They’re not going to eat.

Yeah, if the morning is bad, the whole day is bad.

Small choices, such as when they wake up and when or what they eat, what activities they participate in, and who works with them, are all ways to increase power and activity. Although staff do not use social science terminology to explain their strategies, they do, nonetheless, recognize how having choices and attractive options improves mood and encourages participation in the life of the unit.

Such small choices take time and effort on the part of the staff but seem to bear large dividends for residents. Despite substantial cognitive impairment, residents seem largely happy and engaged most of the time—often even getting into mischief (e.g., going through the drawers in the nurses station or trying to boss around other residents). The research team members were often approached by residents wanting to talk to us or tell us something; in confirmation of much of what we heard from staff, without background knowledge of the resident, we frequently had difficulty understanding what he or she was trying to communicate.

Families understood this as well, identifying the continuity of having the same staff who know the residents well as being one of the key reasons their family members were happy at Twin Elms. One daughter of a resident went into eloquent detail about the importance of this:

Yeah the continuity. The same nurses and the same nurses’ aides cause when she first moved to that room that was an overflow room. She did not really need to be there [in the dementia wing] in the last unit and I asked the head nurse there, Connie,… “what are the chances mom goes in here she can go back on the other wards at some point” she goes “well I’ll tell you a story” she says “we have had many patients go to overflow over there and then go back to the regular hall and then want to go back” and she said to us because I have had the same staff in there for over 20 years. So, there are some nurses’ aides in their Marilyn and Abigail there over 20 years. One of them is like 27 years. So, mom talks about them all the time and I’ll say “oh let me talk to her on the phone” she’ll hand them the phone and I’ll talk to them “hey is mom get low on her hair shampoo or whatever” and so I’ve gotten quite a rapport with them even though it’s telephonic and mom just loves them there. When they are not there she goes “O I got a crummy one today” I go “what do you mean are they mistreating you?” “Oh no they are just not as nice as Marilyn and Abigail.” “I suppose that’s ‘cause they know you mom.” So, they can get her pretty much to do anything. Although one day they’d ask her, ok Monday’s you get your hair done today “No, I do not feel like it.” “Well what do you mean you do not feel like it?” “I’ve got a headache.” Ok fine. So, sometimes she’ll stand her ground, but most of the time the staff will say “ok well we’ll come back in 1/2 an hour” and then “ok take your Tylenol” and then she’ll go to take her Tylenol and then she’ll go. So, staff that knows how to work with those that have been there quite a while. It’s like you know, they call me if something’s going on or whatever and it’s like mom knows some of them by name, but they have been floating some other ones there from the other side when they are short and she is getting some of the same ones, but you can tell there’s that. The thing is the continuity that’s what helps a type of patient like my mom (I: Well what we are trying to do with this app is…help staff who do not work with those particular residents often). Oh that’s helpful! Yeah, because a couple of the ones that said, when I’ve had them on the phone, “well we do not normally work down here we are doing our best.” I said, “do not worry hang in there you are doing good” They have had people floating that have not had training.

Many people do not have the luxury of getting to know residents the way that regular staff on the dementia wing at Twin Elms have done. As reported by both families and staff members, unfamiliar staff (such as floaters) struggle with knowing the best way to interact with residents, especially those who can no longer express themselves well. Even relatives and friends of the resident, if they have not been regular visitors, can find themselves at a loss because their past knowledge is not enough. The goal of the VIPCare application, therefore, is to try to address some of these gaps in knowledge and provide unfamiliar individuals with strategies to interact more comfortably and effectively with a person with dementia.

One cellphone application, of course, cannot pretend to be a substitute for true knowledge and the time and effort of getting to know a person. Our goal, however, is to provide some short cuts that can facilitate learning how to interact and help avoid negative and off-putting events. A person who finds that all they do is upset a resident is unlikely to visit very often—which is itself a negative outcome. By programming the application using EPA dimensions and resident self-sentiments, we seek to simulate ways to interact with residents that will not lead to identity disconfirmation and the disruption of interaction.

### 3.3 Results: the VIPCare application

As discussed in the introduction to this paper, VIPCare is based on the premises of Affect Control Theory. ACT frames interactions as Actor-Behavior-Object (ABO) events that generate transient impressions—that is, impressions on the sentiments of the Object person, in this case, the resident. If those impressions do not agree with (or confirm) the self-sentiments of the resident, that object person will experience an uncomfortable feeling, called a deflection. In usual interaction, the object person is motivated to respond in ways that bring the transient impressions into better agreement with their own sentiments. However, people with dementia have limited interactional resources. As Francis et al. (2020) found, people with cognitive decline gradually lose their ability to recognize the cognitively based denotative cues (MacKinnon, 2015) needed to define situations. They therefore cannot access the shared definition of the situation and so cannot actively manage the interaction to reduce the deflection. Expression of emotion resulting from the deflection from usual sentiments (e.g., agitation, anger, sadness, humiliation, etc.) is the most likely outcome.

**Figure 1:**
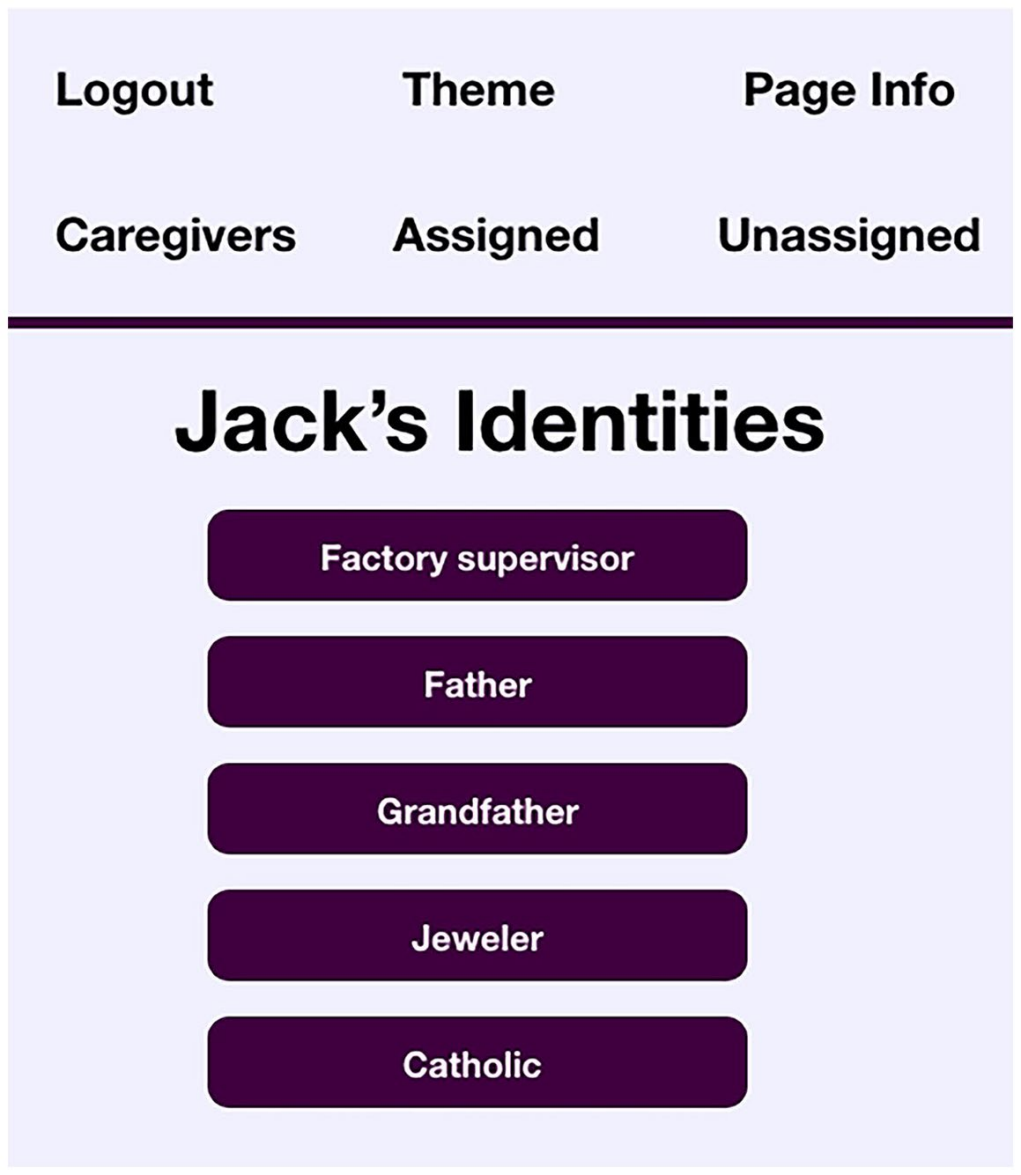
VIPCare screenshot identities of resident “Jack”.

VIPCare serves as a prompt to inform the cognitively intact individual (e.g., floater, visitor, etc.) of what kind of self-sentiments the resident most likely harbors. Human beings are often quite good at ascertaining the proper response to another person’s role or identity, if they know what it is.^2^ The setup of the application, therefore, first requests the creation of a resident profile containing the most valued identities of a resident. This is in the form of a basic interface where the user selects identities from an alphabetical list drawn from the 2015 ACT dictionary (Smith-Lovin et al., 2016) and is uncomplicated enough for lay users like caregivers. This populates the probability distribution of potential identities on which the resident is most likely to draw upon in any given interaction (Schröder et al., 2016). As mentioned earlier, the caregiver is then assigned a complementary (non-deflecting) identity for the purposes of the initial interaction. Figure 1 shows Jack’s list of identities in the application.

This is not an exhaustive list of Jack’s potential identities, but rather reflects those that family members viewed as the one’s having the greatest impact on Jack’s self-sentiments. Although most people have potentially hundreds of identities within their repertoire that they can enact when salient to situations they encounter (MacKinnon and Heise, 2010), the limited access to shared definitions experienced by people with dementia means that their repertoires of identities shrink with the progress of the disease. Once entered, the screen of resident identities does not show again unless it needs to be edited.

**Figure 2:**
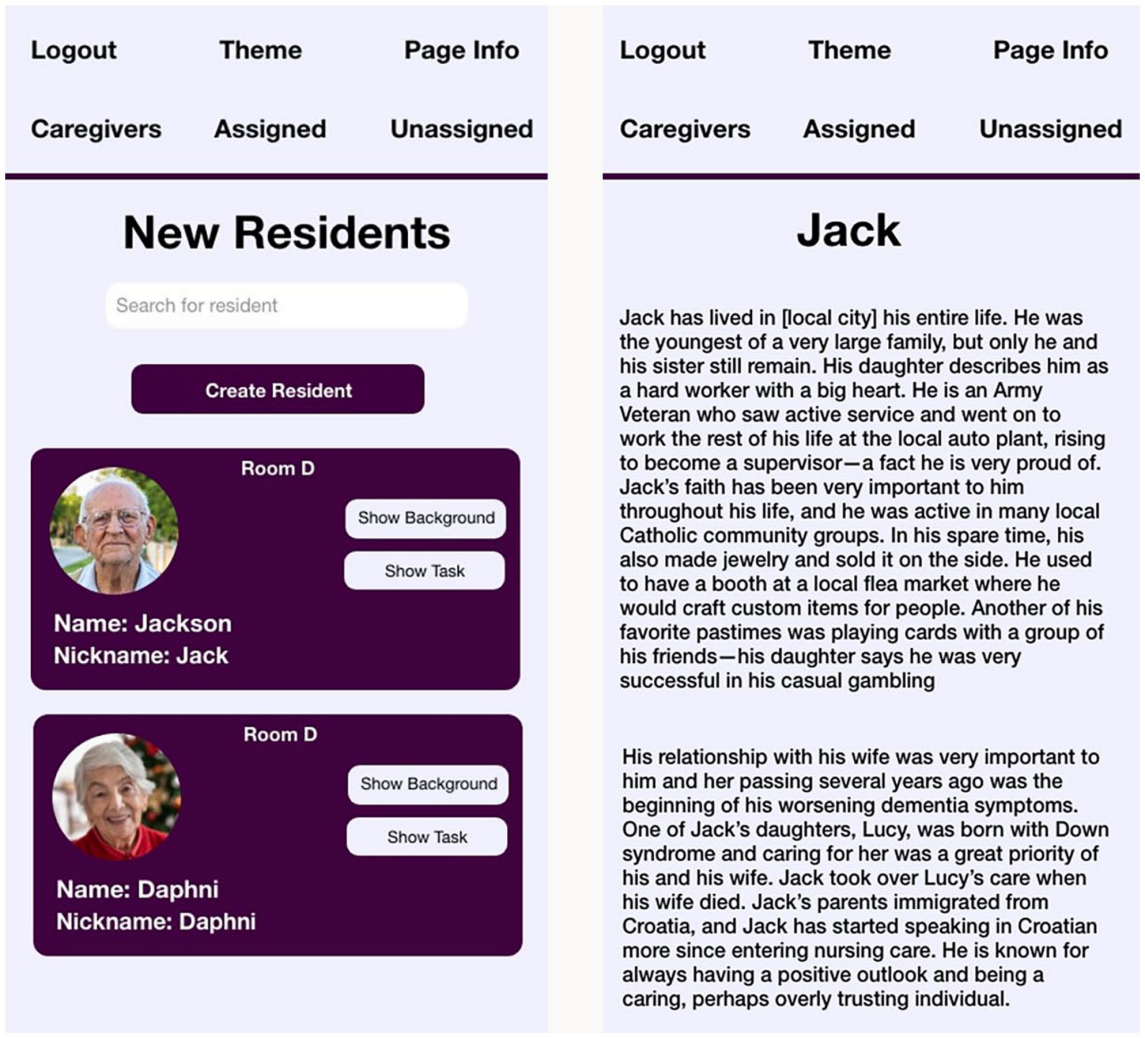
Home and background screens of VIPCare application.

For users of the application (labeled caregivers in VIPCare, as those were the targets of the current study), the first step in using VIPCare is to select the resident. For floaters, new staff members, or others unfamiliar with the resident’s history, the screen with the list of residents has not only the photo, name, nickname (if relevant), and room number of each person, but also two buttons. The Background button leads to a brief biography of the resident, modeled after the shadowboxes that families sometimes create and hang on the door outside their family member’s room at Twin Elms. The opening screen is shown in Figure 2 (photos are stock photos, and names and background details have been modified for confidentiality).

Two residents are shown, Jack and Daphni, who were profiled above. Staff members can both use the search bar to find residents by name or room number, or they can scroll through to choose residents by photo.

To activate VIPCare, a user taps on the second button (Show Tasks) on the opening screen shown in Figure 1, revealing the second screen (see Figure 2). On this screen, the user selects the task or behavior for the situation. These tasks were chosen by the Twin Elms staff members in the co-design focus groups (Ghafurian et al., 2022).

**Figure 3:**
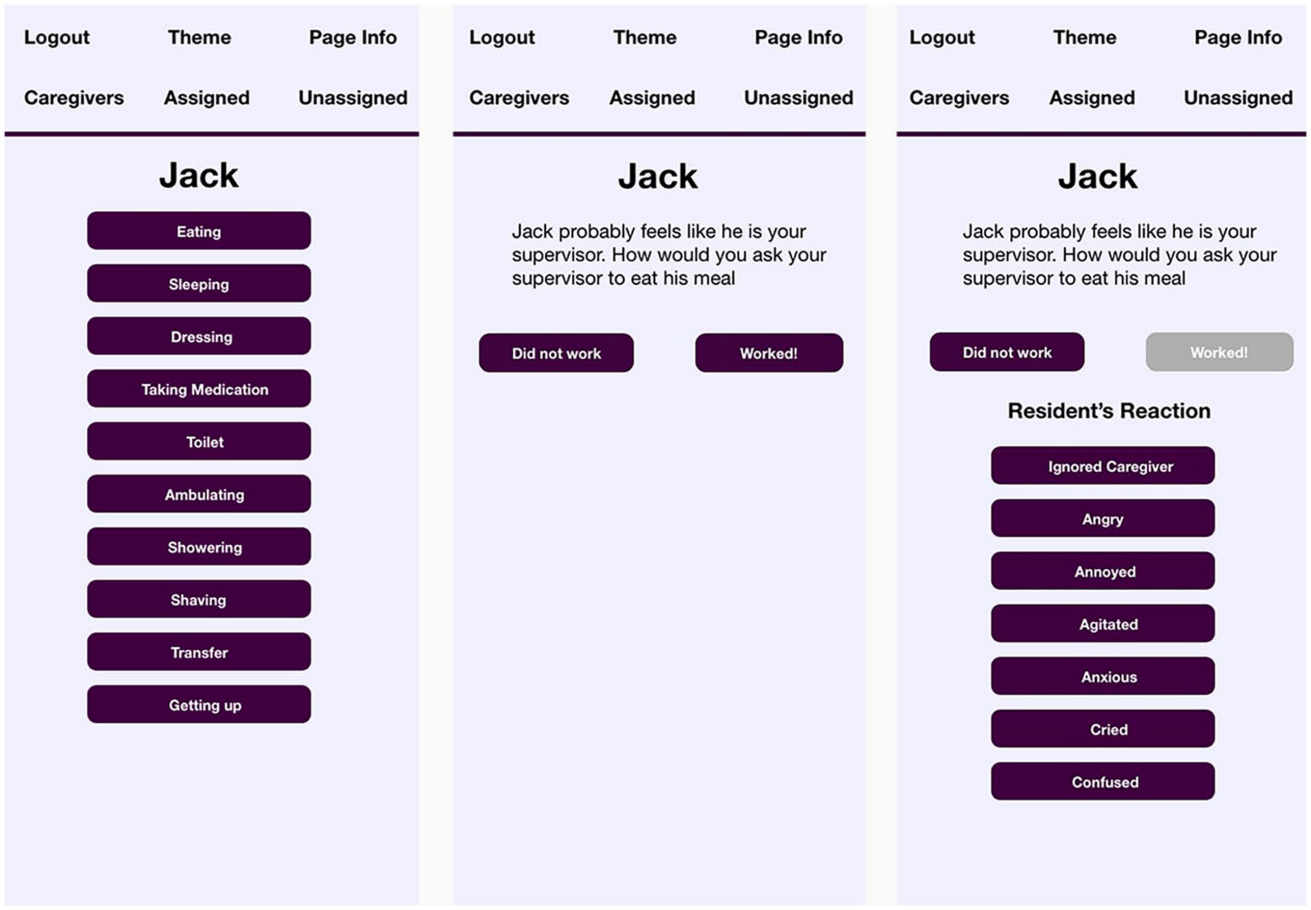
Task, suggestion, and reaction screens of VIPCare for resident “Jack”.

VIPCare then constructs an Actor-Behavior-Object person event by drawing on the sentiment profile of the resident to select the most probably identity for that person and populate the staff role out of corresponding identities. VIPCare will automatically open the third screen, where, based on the EPA rating of that identity, VIPCare then suggests an approach to interaction that will confirm the resident’s self-sentiments. Screens two and three (and four, discussed below) are shown in Figure 3.

As screen two (the first screen in Figure 3) shows, Jack’s identity as a supervisor at the auto plant is the most probable one. It is very common for men with dementia to prioritize the work identity—especially in the pre-WWII generation represented in this study—and they often view the facility as their workplace and staff and other residents as employees. Among men in leadership positions at work, one would expect this to be even more pronounced because of the value of the identity. VIPCare, drawing on Jack’s sentiment profile instructs the user that the best way to avoid deflection for Jack is to treat him as though he is the user’s supervisor. This is what Jack expects to be at work, and he is likely to respond well to that approach.

The equations underlying Affect Control Theory predict that the best strategy to approach one’s supervisor is to ‘request,’ ‘ask,’ or ‘entreat.’ Thus, the suggestion programmed into VIPCare reflects that approach with the statement, “How would you ask your supervisor to eat his meal?” The open-ended phrasing (‘how would you’) taps into the ability of humans to use their own agency to adapt to situations and respond creatively to novel events (Smith-Lovin and Heise, 1988).

Note, however, that the suggested identity is based on a probability analysis and there is a non-negligible chance of error, meaning that Jack is not enacting his work identity. In the final screen of Figure 3, the user has tapped the button “Didn’t work,” and the list of reactions appears. These reactions were compiled from staff member input in the co-design process. If Jack reacts by ignoring the caregiver (not a typical reaction of a boss to a request from an employee), VIPCare engages artificially intelligent computer-learning based on Bayesian analysis to select another identity from Jack’s sentiment profile that coheres with the EPA ratings of the behavior “ignore.” Thus, for example, a supervisor is unlikely to ignore a requesting employee, but a father may ignore a begging child without causing serious deflection. VIPCare, therefore, might posit that Jack feels that he is a father and the staff member a beseeching child, and it will suggest a different behavior based on the transient impressions of that ABO event. That suggested behavior will be calculated to restore deflected impressions to the established sentiments of a father, thereby confirming Jack’s self-sentiments and supporting his self-concept and mood. By way of benefit to the staff member, such a change will also likely make Jack more cooperative and amenable to future suggestions.

**Figure 4:**
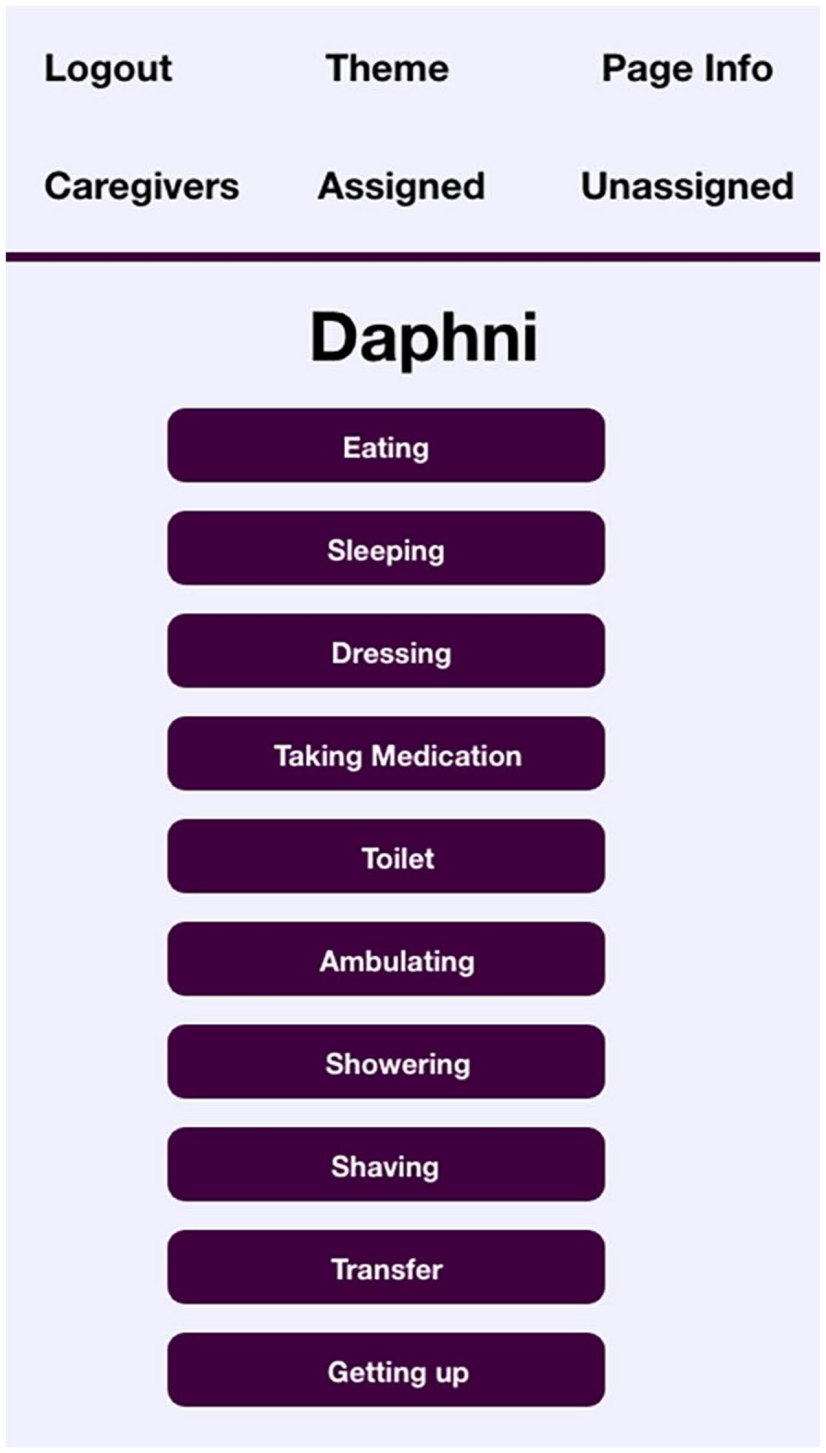
VIPCare screenshot identities of resident “Daphni”.

By way of contrast, the screen showing identities entered for the profile of resident Daphni are shown in Figure 4.

**Figure 5:**
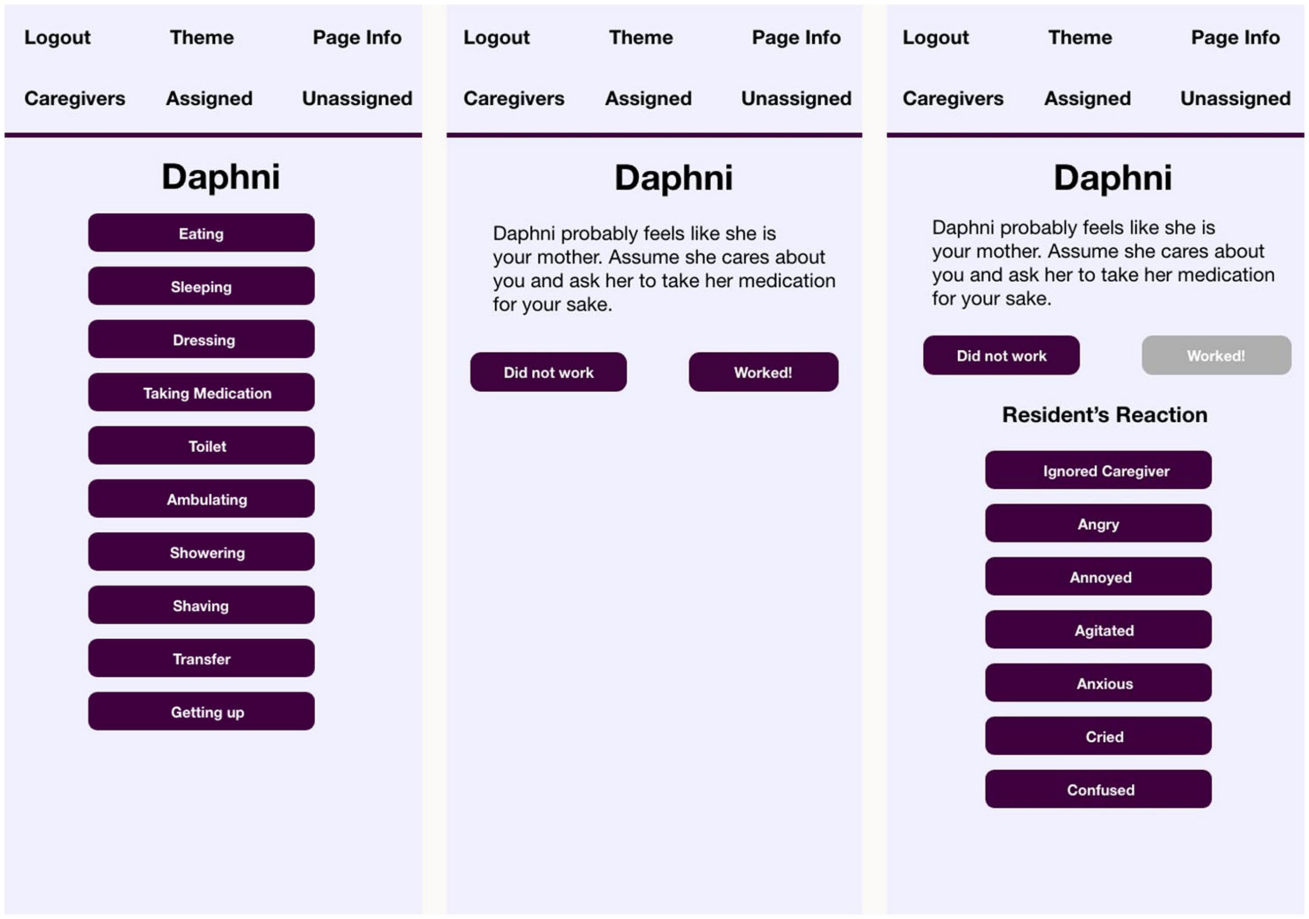
Task, suggestion, and reaction screens of VIPCare for resident “Daphni”.

Daphni, though an immigrant from a farming community in Greece, has her most recent identities organized around her roles as a wife and a mother. Even her occupational choices (Cook) are in keeping with the nurturing and caring behaviors that her daughter describes as typical of her. Daphni, therefore, may be likely to view herself as a *mother* in her interactions, and VIPCare selects that is the most probably identity, as shown in Figure 5.

From these two examples, Jack and Daphni, we can see how VIPCare proffers different identities for different residents: work supervisor and mother. These are common identities for the generation of these residents, which tended toward traditional gender roles. Most people trying to interact with Jack and Daphni would expect them to have these identities. However, what is unique about VIPCare is that it helps the user recognize that Jack and Daphni are likely to enact these identities *even when the situation calls for other ones*. As a result, their behavior and reactions may be out of sync with the expectations of a person who is drawing from the immediate perceived situation for their cues, rather than from the history and biography of the person with dementia. The application then prompts the user to engage with Jack or Daphni in the identity that the resident is using, rather than as their perceived identity (e.g., father, friend, sister-in-law, patient, nursing home resident). Asking Jack if he’s getting good care, therefore, might confuse him, because that is not something that work supervisors are generally asked. Daphni, as a homemaker, might respond better to that question: the EPA rating of a mother identity is less powerful and active, but more good than work supervisor, therefore more deserving of help. However, we are reminded by the interview quote from Daphni’s daughter that mothers in their home territory are also prone to feel that they should be in charge. So indicating to either resident that they might not be self-sufficient, a.k.a., treating them as patients, or nursing home residents, or similarly weak, passive, and not very good identities, will likely disconfirm their self-sentiments. VIPCare directs the user to try to maintain impressions of events that support the resident’s self-sentiments, thereby helping to preserve the self the resident has always had.

## 4 Discussion

This paper has reported on the use of Affect Control Theory for the development of a cellphone application designed to facilitate effective interaction between users (e.g., family members, professional caregivers, visitors) and persons with late life dementia in a residential nursing facility. VIPCare is unique in being one of a very small number of applications based on and built out of established sociological theories of human interaction. Sociological theory, in general, is not widely used in most applied disciplines, and that has left a gap in how problems of social life are addressed. Computer applications, which depend on human behavior, are a key place of overlap between theory and practice.

Beginning with the theoretical predictions of Affect Control Theory, the study team started with staff focus groups, seeking open-ended responses to how they interact effectively with the residents on the dementia wing. Results confirmed the expectations of ACT, underscoring that knowing a resident well—especially their past identities and the kinds of roles they held—was the most important information. Such knowledge set the stage for being able to work with residents in ways that “keep them happy,” prevent them from “getting combative,” and “help them stay engaged.” These groups also produced the design and format of the application.

Interviews with family members, those who cared for the residents before they moved to Twin Elms, also confirmed the findings from the groups. “Continuity” and “knowing” the residents well were key to working with them successfully, and part of why the staff of the wing were so loved by families. Interviews also provided the identity information needed to construct the sentiment profiles. The resulting VIPCare application can thus be tailored to the self sentiments of each individual resident and provides prompts to interact with residents in ways that confirm those sentiments.

Following the premises of ACT, such support of resident self-sentiments should support and help preserve the sense of self they have had their entire lives. This, while not a treatment that improves dementia, has at least the potential of improving the quality of life of people who have cognitive impairment. By reducing deflection and the negative consequences of repeated disconfirmation of self sentiments, the application has the potential of preventing distress before it occurs. This, in turn, may reduce the need for the psychotropic medications commonly used for agitation, depression, and behavior problems among people with dementia (Ramadan et al., 2003).

A key contribution of this paper, then, is the reporting of a translational study, in which a sociological model of human interaction and affect is incorporated into a tool to be used by those on the front lines of dementia care. Staff and family members who are the likely users of the application informed and supported the theoretical model. This is forging into relatively new territory for sociology, which often has been prone to producing basic research read only by other sociologists.

VIPCare is a work in progress. Progress so far has identified several gaps that need to be addressed in future research. First, as mentioned in the description of the sentiment profiles, we plan to add modifiers about the *type* of person or identity; these adjectives will add greater depth to the sentiments associated with that identity, and thus, greater precision to the simulation of the interaction. Annie might become a *quiet sister*, which would dampen the activity rating of her predicted reactions. On a related note, we used the most recent ACT dictionary available to populate VIPCare, and this may not be the best approach. Cultural meanings of identities change slowly, but they do change (Heise, 2019). ACT has dictionaries going back decades and leveraging meanings from dictionaries collected earlier when the residents were active in their identities may provide more accurate predictions; this is something to be explored as we further develop VIPCare.

Second, we plan to create likely role sets (Merton, 1960) for each identity, such that Daphni might be *a* mother, but not necessarily *your* mother in the interaction. For non-insitutional identities (non-role-identities) such as “hard worker,” further research will be needed to determine if duplicating that identity for the actor is the best strategy, or if we need to ascertain the least deflecting identity for each such case. The latter would not be ideal, as a case-by-case evaluation would make the application less accessible to lay users. Currently, VIPCare’s first screen is a simple initial interface that allows anyone with a username and password to enter identity information (eventually including modifiers such as traits or characteristics). The issue of how to program counter identities for the caregiver will need to be resolved to keep that interface as manageable as possible.

Related to this is a third question on how much framing should the application do for the user? ACT does have the ability to propose actions that are non-deflecting, but they are largely generic to identities and may not always be appropriate to the “real life” situation. A resident may feel like a grandmother to a staff member, but appropriate actions for a grandchild include behaviors like kissing and snuggling—which may be against the rules for staff members. To address all of these concerns about identities and suggested framings will require a larger and more varied sample.

Finally, we will need to evaluate the application in practice to see if users (and which users) find it helpful. Challenges in this study underscore that to get more accurate data on the self-sentiments of persons with dementia, we need to refocus our attention on individuals who are in earlier stages of their disorder than most residents in nursing facilities. While the relatively controlled conditions and limited identities in an institutional setting were an appropriate first site, greater variation will be informative in next steps. Thus, persons still living in the community with their family caregivers will be our next prospective research participants to further develop the application.

This study has several limitations as well. First and foremost, the data collection began in the second half of 2019. The March 2020 interruption of the pandemic put residential nursing facilities into full quarantine, which truncated data collection and reduced the sample size. Second, the two-year delay resulted in the loss of many participants: frail residents died (though not of COVID in this well-run facility), and many staff left during high pandemic turnover. Thus, evaluation of the application’s functioning will require a new data collection effort with current staff, residents, and families. Third, the research site in this study was a middle-class facility with mostly white residents. Future research needs to include residents of more diverse facilities, which are also often those with fewer resources and lower ratings. The problems of staff turnover and psychological distress among residents are likely to be more prevalent in such settings, so ensuring that VIPCare responds to the diversity of residents and staff is essential.

## 5 Conclusion

The project reported here is quintessential translational research, applying theory and technology from the academy to the practical needs of dementia care. The social scientists on the research team will develop these findings to further understand the role of affective meaning and interaction in psychological distress among people with dementia. The results of this study also will have a direct impact on the work being done on the design and development of automated technologies for the support of caregivers of people with dementia. These devices will be able to build on the insights from this study to provide more effective and engaging assistance, helping to preserve the self of people struggling with the existential threat of cognitive decline.

## Data availability statement

The raw data supporting the conclusions of this article will be made available by the authors, without undue reservation.

## Ethics statement

The studies involving humans were approved by Cleveland State University Institutional Review Board. The studies were conducted in accordance with the local legislation and institutional requirements. The participants provided their written informed consent to participate in this study.

## Author contributions

LF: Conceptualization, Data curation, Formal analysis, Funding acquisition, Investigation, Methodology, Project administration, Resources, Supervision, Writing – original draft, Writing – review & editing. MG: Data curation, Formal analysis, Investigation, Methodology, Software, Writing – original draft, Writing – review & editing.

## Footnotes

^1^U.S. 2015 dictionary.

^2^Personal communication, Lynn Smith-Lovin, American Sociological Association Meetings, 2023.
